# Detection of A-to-I RNA Editing in SARS-COV-2

**DOI:** 10.3390/genes13010041

**Published:** 2021-12-23

**Authors:** Ernesto Picardi, Luigi Mansi, Graziano Pesole

**Affiliations:** 1 Department of Biosciences, Biotechnology and Biopharmaceutics, University of Bari “Aldo Moro”, Via Orabona 4, 70125 Bari, Italy; luigi.mansi92@hotmail.it (L.M.); graziano.pesole@uniba.it (G.P.); 2 Institute of Biomembranes and Bioenergetics, National Research Council, Via Amendola 122/O, 70126 Bari, Italy; 3 Consorzio Interuniversitario Biotecnologie (CIB), 34012 Trieste, Italy

**Keywords:** SARS-COV-2, RNA editing, ADAR, transcriptome

## Abstract

ADAR1-mediated deamination of adenosines in long double-stranded RNAs plays an important role in modulating the innate immune response. However, recent investigations based on metatranscriptomic samples of COVID-19 patients and SARS-COV-2-infected Vero cells have recovered contrasting findings. Using RNAseq data from time course experiments of infected human cell lines and transcriptome data from Vero cells and clinical samples, we prove that A-to-G changes observed in SARS-COV-2 genomes represent genuine RNA editing events, likely mediated by ADAR1. While the A-to-I editing rate is generally low, changes are distributed along the entire viral genome, are overrepresented in exonic regions, and are (in the majority of cases) nonsynonymous. The impact of RNA editing on virus–host interactions could be relevant to identify potential targets for therapeutic interventions.

## 1. Introduction

SARS-COV-2 is an enveloped virus with a positive sense, single-stranded RNA (ssRNA) genome of about 30 kb belonging to the genus betacoronavirus [1], sadly known for causing the pandemic by coronavirus disease 19 (COVID-19) [2]. Comparative genomics, from thousands of complete viral sequences of SARS-COV-2 from diverse geographic sites, has revealed a biased substitutional pattern in which the C-to-T change outnumbers in all other substitutions [3]. The non-random occurrence of this mismatch strongly suggests that the SARS-COV-2 genome could undergo C-to-U RNA editing through APOBECs, as shown in metagenomic experiments from bronchoalveolar lavage fluids (BALF) of COVID-19 patients [4]. On the other hand, there are contrasting evidences on the occurrence of A-to-I RNA editing [4,5], even though the A-to-G change appears the second most common mismatch type [3] and a recent study based on 62,000 viral isolate sequences (from human infections in the USA) ranks A-to-G (and T-to-C) changes third among all detected mutations [6].

RNA editing by adenosine deamination is carried out by ADAR enzymes and is prominent in the human transcriptome in which it converts As in Is in long double-stranded RNAs (dsRNAs) formed by repeated elements in opposite orientation (mainly Alu sequences) [7]. Human cells harbor three ADAR genes: ADAR (also known as ADAR1), ADARB1 (also known as ADAR2), and ADARB2 (also known as ADAR3) [8]. ADAR1 and ADAR2 are catalytically active and expressed in almost all human tissues, even though ADAR2 expression shows lower levels than those observed for ADAR1 [9,10]. While ADAR2 tends to edit As in coding protein sequences, and has only been detected in a few instances up to now [11], ADAR1 extensively deaminates As in long dsRNAs and exists in two different isoforms: ADAR1p110, constitutively expressed, and ADAR1p150, mainly located in the cytoplasm and inducible by type-I interferon [8].

Recently, it has been shown that A-to-G changes found in metagenomic sequences from BALFs of COVID-19 patients could be due to the activity of ADARs [4], but strong evidence of A-to-I RNA editing in the SARS-COV-2 genome has not been provided. Indeed, it is well known that ADAR1 tends to edit sites in clusters (hyper-editing) and exhibits a specific sequence context with G depletion one-base upstream of the edited site [7,12]. These two important signatures were not detected in metagenomic sequences that, by their nature, prevent the accurate quantification of ADARs as well as their RNA editing activity at the transcriptomic level. Additionally, a concomitant study describing the transcriptome of SARS-COV-2 in infected Vero cells. by using the nanopore direct RNA sequencing and the DNA nanoball sequencing, excluded ADAR-mediated deamination for the lack of A-to-G changes [5].

ADAR1 has a pivotal role in the modulation of the innate immune response, i.e., the first line defense against foreign viral nucleic acids [13,14,15]. Through proteins called nucleic acids sensors, such as the endosomal Toll-like receptors (TLRs) and the cytoplasmic retinoic acid-inducible gene I (RIG-I)-like receptors (RLRs), typical intermediates of virus replication, such as dsRNA or ssRNA, can be recognized and can induce the production of type-I interferons [16]. In turn, type-I interferons activate the expression of interferon-stimulated genes (ISGs), including ADAR1p150 and members of the APOBEC protein family [17]. Once produced, ADAR1p150 can have antiviral effects by destabilizing dsRNAs through multiple A-to-G substitutions, an occurrence termed hyper-editing, or proviral effects can suppress the innate immune response by A-to-I RNA editing of long dsRNAs [18,19]. Consequently, exploring the origin of A-to-G changes occurring along the SARS-COV-2 genome could be quite relevant to better understand the host–virus relationships or the evolutionary dynamics of the viral genome and identify potential targets for therapeutic interventions.

Here, we prove that A-to-G changes observed in the SARS-COV-2 genome are genuine RNA editing events likely mediated by ADAR1. By using an ad hoc computational workflow to mitigate the noise of sequencing errors, we were able to detect A-to-I editing in human and Vero-infected cell lines as well as in several clinical samples.

## 2. Materials and Methods

### 2.1. RNAseq Data

Raw RNAseq data were downloaded from SRA under the following BioProject accessions: PRJNA625518, PRJNA616446, PRJNA601736, PRJNA605907, and PRJNA631753. RNAseq reads of infected Vero cells were downloaded from the Open Science Framework (OSF) with accession number https://doi.org/10.17605/OSF.IO/8F6N9, while data from uninfected Vero cells were obtained from SRA using the accessions: DRR018832, DRR018833, DRR018834, and DRR018835.

### 2.2. Filtering of RNAseq Raw Data

Raw reads were cleaned using FASTP [20] and the read length was taken into account. For reads longer than 76 bases, we trimmed 10 nucleotide upstream and downstream, and removed reads with more than 20% of unqualified bases (-q 25 -u 20 -l 50 -x --cut_tail --cut_tail_mean_quality 25 --trim_front1 0 --trim_tail1 0). The trimming was disabled for reads shorter than 76 bases. The mean quality per base was fixed at a phred-score of 25. Reads shorter than 50 bases were removed.

### 2.3. Alignment of RNAseq Reads

Cleaned reads were aligned onto a comprehensive reference sequence, including the whole human genome (hg19 assembly from UCSC) and the SARS-COV-2 genome (NC045512.2 from NCBI) by bwa [21] using default parameters. Unique and concordant reads mapped on the SARS-COV-2 genome were extracted by sambamba [22] and converted in BAM format by SAMtools [23]. Viral reads were also aligned onto the NC045512.2 assembly by GSNAP [24] with the transcriptome-guided strategy. SARS-COV-2 transcript annotations were obtained from UCSC. The strand orientation per sample was inferred by the infer_experiment.py script from the RSeQC package [25]. Additionally, human reads were also aligned onto the human reference genome by STAR [26], proving known GENCODE (v31lift37) annotations.

In Vero cells, the human genome was replaced by the *Chlorocebus sabaeus* genome (chlSab2 assembly) from UCSC. Green monkey annotations were also downloaded from UCSC.

### 2.4. Detection of Hyper-Edited Reads

Hyper-edited reads were detected using our custom SubstitutionsPerSequence.py script. It takes input viral reads aligned by bwa [21] in BAM format and filters out reads with a mapping quality score lower than 30, those not properly mapped, as well as those flagged as secondary alignments. It also removes reads carrying insertions or deletions, and those with more than 2 substitutions of the different type. Reads with the same mismatch type are further filtered in a similar way, as described in [12]. Dense clusters of high-quality (phred ≥30) A-to-G (or T-to-C) mismatches are detected as retaining reads, in which the number of A-to-G changes is at least 5% of the read length and discarding reads have too dense A-to-G mismatch clusters (length <10% of the read length) or clusters contained within either the first or last 20% of the read or clusters with a particularly large percentage (>60%) of a single nucleotide. When the aligned region is less than 80% of the read length, reads are also removed.

### 2.5. Detection of RNA Editing at Single-Nucleotide Level

We performed an initial variant calling by REDItools (version 2) [27,28] and the same parameters also used in [4] (-os 4 -q 30 -bq 30 -l 0). Strand orientation was taken into account in samples in which libraries were prepared using strand-oriented kits. To remove the noise due to sequencing errors, we used only concordant reads whose alignments were confirmed by two independent aligners, bwa [21] and gsnap [24]. In addition, we excluded discordant base variants at overlapping regions of read pairs. Low-quality reads, as well as reads not properly mapped or flagged as secondary alignment or carrying indels, were removed as well. We excluded a certain number of positions in the first or last regions of reads depending on the read length (5 upstream and 6 downstream for reads < 100 bases, and 15 upstream and downstream for reads > 150 bases). All sites were removed when the variant nucleotide was not supported by at least 4 reads. In strand-oriented experiments, the variant calling was corrected accordingly. In non-strand oriented experiments, instead, the fisher exact test was used to check strand biases.

In the variant calling, we also excluded positions in single repeats and known viral variants from UCSC. The entire procedure of noise correction was implemented in the corr.py script.

After the noise correction, RNA editing candidates were called at a minimal allele frequency equal to two times the error rate, as estimated by the overlaps of read pairs.

### 2.6. Gene Expression in Cell Lines

Differential gene expression was performed by DESeq2 [29] on featureCounts [30] read counts while FPKM values used in the figures were calculated from raw counts using a python custom script, according to the FPKM formula described in [31]. Genes with an adjusted *p*-value of < 0.05 in DESeq2 output tables were marked as differentially expressed.

### 2.7. RNA Editing Enrichment

RNA editing enrichment was calculated by only taking into account unique A-to-I events detected in hyper-edited reads, according to the definition proposed by [12] in which the editing enrichment is equal to the number of unique A-to-I events in each experiment divided by the expected number. Such expected number was computed by multiplying the total number of A-to-I events (over all experiments) by the ratio of the number of mapped reads in the experiment to the number of mapped reads in all experiments, normalized by the viral load.

### 2.8. Alu Editing Index

The Alu editing index (AEI) providing the ADAR activity at transcriptome level was calculated using the RNAEditingIndexer pipeline, as described in [32]. Differential AEI was assayed by the t-test at a significant level of 0.05.

### 2.9. Quantification of Sense and Antisense Viral Strands

Sense and antisense viral strands were only quantified in strand-oriented datasets only and featureCounts [30] were used to annotate the list of known viral non-overlapping coding regions from UCSC. The percentage of the antisense viral strand was calculated as the fraction of reads mapping on coding sequences projected on the antisense strand over the total number of reads mapping on non-overlapping coding sequences.

### 2.10. Annotation of A-to-I Editing Events

RNA editing events were annotated using ANNOVAR [33] by providing the list of known SARS-COV-2 transcripts from UCSC.

### 2.11. Statistical Analysis

Averages and standard deviations represented on graphs were calculated by a python custom script using the pandas module. All graphs were generated using python and the Seaborn module. Statistical comparisons of AEI values were made by the t-test at a significant level of 0.05 using the ttest_ind function from the Python SciPy module. Pearson correlation between hyper-edited reads and viral reads was carried out using the Pearson function from the python scipy module. Statistical comparisons of gene expression was performed using DESeq2 [29].

## 3. Results and Discussion

**Figure 1:**
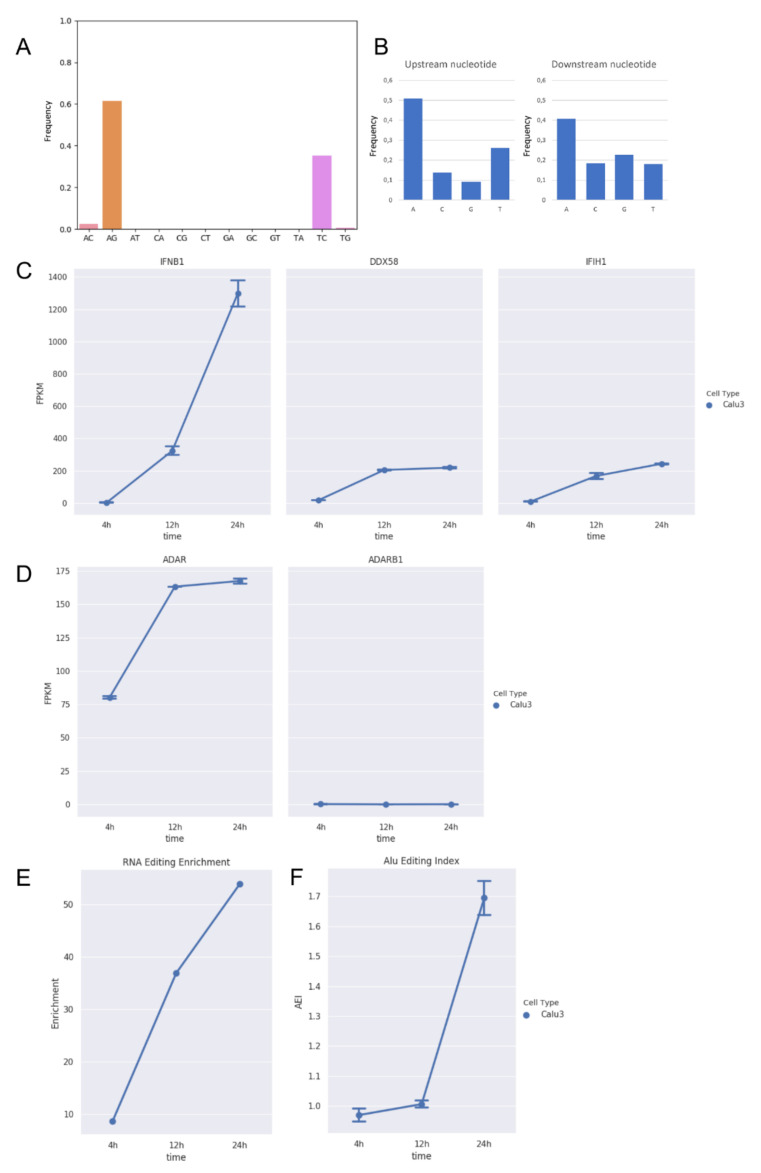
RNA editing and expression of key genes in total RNAseq data from Calu-3 infected cells at three time points post-infection (4 h, 12 h, and 24 h). (**A**) Distribution of hyper-edited reads identified in infected Calu-3 cells in which A-to-G and T-to-C events appear prominent. (**B**) Nucleotide context one nucleotide upstream and downstream the detected hyper-edited sites. (**C**) Gene expression of type-I interferon (IFNB1) and key sensor genes, DDX58 (RIG-I) and IFIH1 (MDA5), in infected Calu-3 cells. Here, we report only IFNB1 as main interferon gene modulated upon infection. As expected, DDX58 and IFIH1 increase their expression during the infection. Gene expression of type-I interferons, RIG-I and TLR receptors, and APOBECs are reported in the Supplementary Figure S1A. (**D**) Expression of ADAR1 (ADAR) and ADAR2 (ADARB1) genes in infected Calu-3 cells. While ADAR2 is expressed at extremely low levels and does not change during the infection, ADAR1 is positively modulated and its expression increases significantly (DESeq2 adjusted *p*-value < 0.05) from 4 to 12 h post-infection. (**E**) Enrichment of unique hyper editing positions in infected Calu-3 cells. (**F**) Alu editing index (AEI) in infected Calu-3 cells. It is a reliable score to measure the ADAR activity at the transcriptome level and tends to grow with the increase in ADAR expression. Dotted lines and bars on each dot indicate mean gene expression or AEI ± SD.

We initially analyzed strand-oriented paired-end reads of data from the total RNA of Calu-3 human epithelial lung cancer cell line infected by SARS-COV-2 at a MOI of 0.3 [34]. Total RNA was extracted at different time points post-infection (4, 12, and 24 h). Viral load was estimated as the fraction of reads mapping on the viral genome over the total number of reads per sample. Raw reads were cleaned to remove low-quality regions and mapped on a comprehensive reference sequence, including the whole human genome and the SARS-COV-2 genome (NC045512) by bwa. Unique and concordant SARS-COV-2 paired-end reads were individually explored and filtered to detect reads carrying high-quality A-to-G clusters (phred-score > 30). In all examined samples, we found a variable number of hyper-edited reads with a significant enrichment toward A-to-G and T-to-C clusters (on opposite strand) (Figure 1A).

While the majority of them were located on the sense strand, only a few A-to-G and T-to-C clusters were observed on the antisense strand, suggesting that A-to-I editing should mainly occur in long dsRNAs during the viral replication. On the whole, we detected 377 unique A-to-G events in 148 hyper-edited reads, and their sequence context showed G depletion one base upstream and a slight G enrichment one-base downstream of the editing sites, strengthening the evidence of ADAR-mediated RNA editing (Figure 1B).

Recent evidences in respiratory epithelial-derived cells and cardiomyocytes infected by SARS-CoV-2 have shown that the virus can induce double-stranded RNA-mediated immune responses [35], leading to the activation of type-I and -III interferons. During the infection, we observed an increased expression of typeI interferon (IFNB1) (Figure 1C, Supplementary Figure S1A) and ADAR1 (Figure 1D), especially at 12 h post-infection (DESeq2 adjusted *p*-value < 0.05), and an enrichment in hyper-editing events (Figure 1E). ADAR2 expression, instead, did not change along the infection.

The hyper-editing enrichment was marked at 24 h post-infection in which we registered a significant increment of the ADAR1 activity measured by the Alu editing index (AEI) (*t*-test *p*-value < 0.01) [32] (Figure 1F), indicating that ADAR1 could be the main player of the observed A-to-I hyper editing and likely through the action of ADAR1p150, an isoform known to be inducible by the interferon [8]. Recent investigations aim to characterize the interactions between SARS-CoV-2 viral RNAs and host cell proteins during infection, whereby ADAR1 appeared as a potential protein interacted with viral RNAs [36].

**Figure 2:**
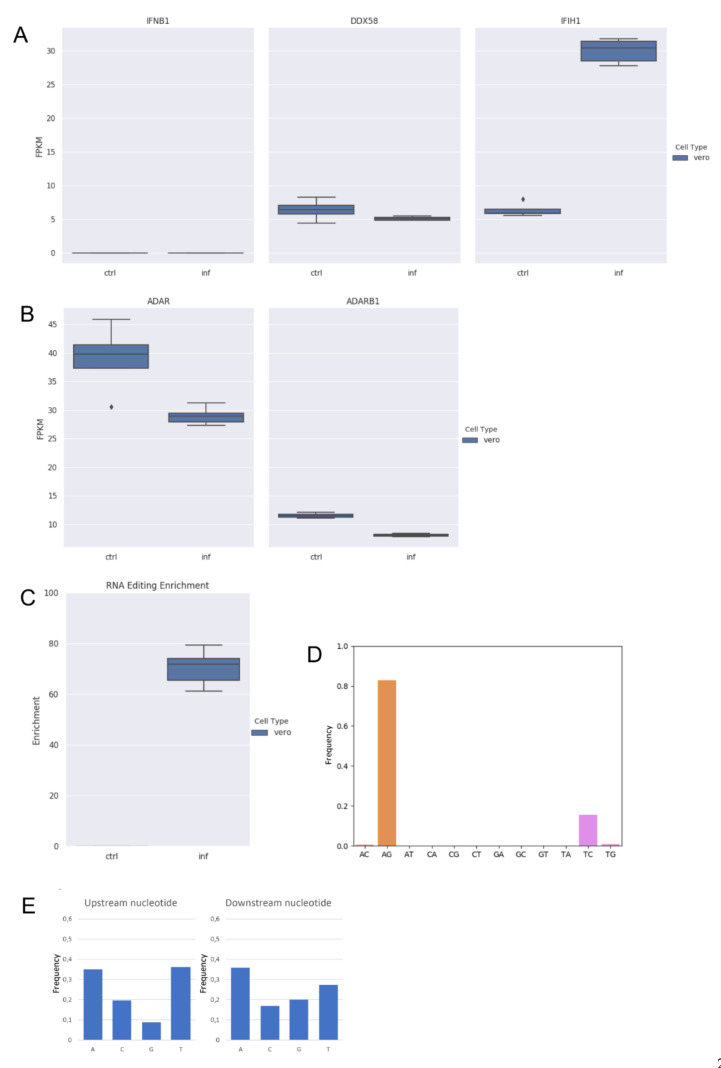
RNA editing and expression of key genes in RNAseq data from infected (inf) and uninfected (ctrl) Vero cells. (**A**) Gene expression of type-I interferon (IFNB1) and key sensor genes, DDX58 (RIG-I) and IFIH1 (MDA5) in Vero cells. Here, we report the expression of IFNB1 as representative gene of type-I interferons. Although IFIH1 were up-regulated, type-I interferons are not modulated. (**B**) Expression of ADAR1 (ADAR) and ADAR2 (ADARB1) genes in Vero cells. Although ADAR1 is downregulated in infected cells (DESeq2 adjusted *p*-value < 0.05), it is expressed in all samples. ADAR2 is also expressed in all samples but at very low levels. (**C**) Enrichment of unique hyper editing positions in Vero cells. (**D**) Distribution of hyper-edited reads identified in infected Vero cells. A-to-G and T-to-C events outnumber other substitution types. (**E**) Nucleotide context one base upstream and downstream the detected hyper-edited sites.

Additionally, we analyzed strand-oriented paired-end reads data from total RNA of uninfected and SARS-COV-2 infected Vero cells in which no A-to-I editing events were detected [5]. Vero cells derive from African green monkey fibroblasts that have lost the ability to produce interferon and are commonly used to grow interferon-sensitive viruses [37]. By comparing RNAseq data of uninfected and infected Vero cells, we initially verified the absence of the type-I interferon response (IFNB1) to the viral infection and the expression of ADAR1 (Figure 2A,B).

Next, by applying the above described computational strategy, we found 1207 hyper-edited reads (~201 per sample) enriched in A-to-G and T-to-C clusters (98% of all hyper-edited reads) (Figure 2C,D), even though ADAR1 appeared downregulated upon the infection (Figure 2B). A-to-I editing was enriched at the same level in all replicates of infected Vero cells (Figure 2C) and the sequence context showed G depletion one-base upstream of the editing sites (Figure 2E), indicating that the SARS-COV-2 genome undergoes A-to-I RNA editing in Vero cells too.

We observed a strong correlation (Pearson: R 0.97; *p*-value: << 0.01) between the number of hyper-edited reads and the number of viral reads, justifying the highest number of hyper-edited reads in Vero cells, despite the lack of type-I interferons. Indeed, an average of 10 M of viral reads were used in Calu-3 against an average of 54 M in Vero cells.

A-to-I events occurring in Vero cells could not be due to the cytoplasmic long form of ADAR1 because of the absence of interferon. As a consequence, observed A-to-G changes could be explained by the activity of ADAR1p110, even though predominantly nuclear [38]. Such a short ADAR1 isoform has actually been shown to be active at a cytoplasmic level in stress conditions [39], which could be induced by the infection, as in the case of Vero cells.

RNA editing and the activity of ADAR1 are tissue- and cell-type-dependent as well as the type-I interferon response to the viral infection [9,40]. To investigate A-to-I editing in different SARS-COV-2-infected cell lines, we analyzed stranded PolyA+ single-end RNAseq data from Calu-3, Caco-2 and H1299 human cell lines infected by SARS-COV-2 at a MOI of 0.3 and generated at three time points (4, 12, and 24 h) post-infection [34]. The viral infection activated the type-I interferon response in Calu-3 cells only and, consequently, ADAR1 did not appear deeply up-regulated in Caco-2 and H1299 cells, as also attested by the AEI index measured at all time points (Supplementary Figures S1B–E and S2A–C). We found A-to-G and T-to-C hyper-edited reads only in Calu-3 and Caco-2 cells but the total number of edited reads was quite low as a result of the PolyA+ sequencing strategy in which mature viral transcripts rather than full genomic RNAs are captured. Additionally, viral reads from PolyA+ data were about four orders of magnitude less abundant than total RNAseq data.

In parallel, we profiled RNA editing at single-nucleotide resolution using the strategy described by Di Giorgio et al. [4] but implementing more stringent filters. We used only concordant paired reads whose alignments were confirmed by two independent mappers (bwa and gsnap). Single-nucleotide variants detected by REDItools [27] were called at an allelic fraction which was two times higher than the error rate estimated by the overlap of read pairs. Strand biases were corrected by employing the strand-oriented protocol for sequencing. In Calu-3 total RNA data, we found 756 putative A-to-I events, increasing from 4 h to 24 h post-infection, and accounting for about 35% of all nucleotide variants. Interestingly, about 42% of all base changes were C-to-T substitutions most likely due to the APOBECs activity. In infected Vero cells, we detected 741 A-to-I candidates but we did not observe an enrichment in A-to-G and T-to-C events. As in Calu-3 cells, C-to-T changes outnumbered the majority of inferred substitutions even though the G-to-A change emerged as prominent. In PolyA+ data, instead, only a tiny number of nucleotide variants was detected and again C-to-T appeared the most representative substitution.

As already shown in [4], A-to-I candidates as well as C-to-U candidates displayed very low editing levels (less than 1% in more than 99% of positions). Furthermore, Alu repetitive elements in the human transcriptome tend to be edited at extents lower than 1% [32,41] which strengthens the idea that ADAR1 should be the main player of the SARS-COV-2 adenosine deamination. However, differently from sites in hyper-edited reads, events detected by this strategy should be regarded with high care. While in the human transcriptome, A-to-G changes due to RNA editing can be distinguished from SNPs by means of whole genome (WGS) and/or whole exome (WES) sequencing data [42]. In the SARS-COV-2 RNA genome, this distinction cannot be achieved. Although RNA editing modulation observed in time-course experiments is a remarkable evidence, genuine RNA editing substitutions cannot be easily discerned from nucleotide variants due to sequencing or polymerase errors.

Finally, we re-analyzed metagenomic samples already used in [4] but limited our workflow to samples in which only paired-end reads were available (Table 1 and Supplementary Table S1).

In BALF samples from the BioProject PRJNA605907 [43], we only found hyper editing enrichment in experiments with a high number of viral reads (>4.5 M). Such samples showed a deep coverage depth of the viral genome (also > 7000×) and more than 75% of detected substitutions were A-to-I candidates. In the two metagenomic BALF samples from BioProject PRJNA601736, we were unable to identify hyper-edited reads, and only a few putative RNA editing sites were detected at a single-nucleotide level. In these samples, however, the coverage depth of the viral genome was relatively low (103× and 430×) as well as the number of viral reads (~65,000 on average).

We also analyzed RNAseq samples from BioProject PRJNA616446, including reads from BALFs and throat swabs. We detected a few hyper-edited reads only in BALF samples and the distribution of nucleotide variants was in line with previous observations from metagenomic samples. Viral genomes from throat swabs were supported by a low number of reads. We also tried to detect RNA editing in RNAseq experiments from post-mortem human donors of the BioProject PRJNA631753, but the number of viral reads per sample was too small to infer high-quality A-to-I events.

Together, our results from infected cell lines and clinical samples show clear A-to-I editing signatures in the SARS-COV-2 genome, even though its reliable profiling is strictly dependent on the sequencing strategy and the number of viral reads (in turn related to the viral load). In all cases, the impact of ADAR-mediated RNA editing on the SARS-COV-2 genome, in terms of A-to-I events or hyper-edited reads as well as editing frequency, is generally low, most likely due to the following factors: (1) the absence of very long dsRNAs along the viral genome or subgenomic regions that could be firmly bound by ADAR1 [44]; (2) dsRNAs from intermediates of viral replication, that are expected to be the optimal targets of ADARs, are poorly represented, and the antisense strand is less than 1% as abundant as the sense counterpart (estimated by stranded RNAseq data); (3) viral RNA synthesis is associated to double-membrane vesicles, preventing the action of cytoplasmic enzymes [45]; (4) the viral RNA-dependent RNA polymerase has proofreading activity that could mitigate the effect of deamination [46]; and (5) SARS-COV-2 seems to have mechanisms to evade and suppress the interferon response, leading to low induction and expression of antiviral genes (including ADARs and APOBECs) [47].

**Figure 3:**
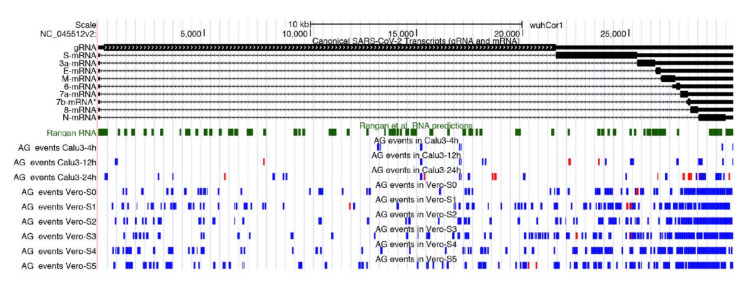
Genomic context of A-to-I RNA editing clusters detected in total RNAseq data from infected Calu-3 cells and Vero cells. We report also known SARS-COV-2 transcripts and putative RNA secondary structures from Rangan et al. [44]. A-to-I clusters in forward orientation are marked in blue, while clusters in reverse orientation are in red.

By taking all unique A-to-I editing events detected in hyper-edited reads of all analyzed cell lines, and representing the high-quality fraction of edited SARS-COV-2 sites, we discovered that they are distributed along the entire viral genome with an apparent preference towards the 3′ end region (Figure 3). This suggests that genomic dsRNAs are, indeed, the main SARS-COV-2 double-strand structures targeted by ADARs.

Furthermore, 96% of sites from hyper-edited reads reside in exonic viral regions and comprise 64% of nonsynonymous events that could have a strong functional impact on the SARS-COV-2 pathogenicity. All annotated sites are available in Supplementary Table S2.

We cannot establish how the virus escapes the antiviral action of RNA editing but several events are fixed and maintained. Although RNA editing occurs at low extent, it could be one of the most relevant mechanisms, governing the dynamics of viral evolution and several studies have already reported evidences in this direction [6,48]. In addition, edited variants could significantly influence the virulence, pathogenicity, and host response. Since the virus tends to evade the RNA editing action, it could have a strong impact on its survival. On the other hand, RNA editing is emerging as a promising therapeutic alternative for different human genetic disorders [49,50] and, thus, it could have an important role in the antiviral fight against the SARS-COV-2 and/or other RNA viruses.

## 4. Conclusions

RNA editing plays an important role in the human immune response to viral infections. Although recent findings from COVID-19 patients and SARS-COV-2-infected cells revealed contrasting evidence and questionable results, we prove that ADAR1-mediated RNA editing in SARS-COV-2 is real and not due to technical artifacts. RNA editing could be a relevant mechanism governing the dynamics of viral evolution, affecting virulence, pathogenicity and host response. Although further investigations are needed to assess the physiological role of RNA editing in SARS-COV-2, detected variants could be important to identify potential targets for therapeutic interventions.

## Figures and Tables

**Table 1:** RNA editing in clinical samples. Here, we report the total number of RNA editing events found at single-nucleotide levels as well as the number and fraction of hyper-edited reads. For each sample, we include the BioProject and run accession, the sampling source, the virus load and genomic depth, the fraction of A-to-I and C-to-U RNA editing events, and the number of hyper-edited reads. Further statistics are in Supplementary Table S1. # This character means percentage of hyper not A-to-I reads.

BioProject	Run	Source	Virus Load	Depth	RNA Editing (All Events)	% A-to-I	% no A-to-I	% C-to-U	% no C-to-U	# Hyper A-to-I Reads	# Hyper non A-to-I Reads	% Hyper A-to-I Reads
PRJNA616446	SRR11454606	Throat swab	0.03	9.64	0	0.00	0.00	0.00	0.00	0.00	0.00	0.00
PRJNA616446	SRR11454607	Faeces	0.24	62.53	1	100.00	0.00	0.00	100.00	0.00	0.00	0.00
PRJNA616446	SRR11454608	Throat swab	3.27	610.55	21	57.14	42.86	38.10	61.90	0.00	0.00	0.00
PRJNA616446	SRR11454612	Sputum	0.08	13.76	0	0.00	0.00	0.00	0.00	0.00	0.00	0.00
PRJNA616446	SRR11454613	BALF	8.33	2256.34	1836	89.43	10.57	8.66	91.34	4.00	0.00	100.00
PRJNA616446	SRR11454614	BALF	18.39	4167.78	6270	84.74	15.26	13.11	86.89	2.00	0.00	100.00
PRJNA616446	SRR11454615	BALF	1.21	321.73	18	50.00	50.00	27.78	72.22	0.00	0.00	0.00
PRJNA605907	SRR11059940	BALF	95.36	21.06	1	0.00	100.00	0.00	100.00	0.00	0.00	0.00
PRJNA605907	SRR11059941	BALF	65.44	1.42	0	0.00	0.00	0.00	0.00	0.00	0.00	0.00
PRJNA605907	SRR11059942	BALF	93.56	478.34	286	89.51	10.49	6.99	93.01	1.00	4.00	20.00
PRJNA605907	SRR11059943	BALF	87.59	39.39	5	0.00	100.00	20.00	80.00	3.00	0.00	100.00
PRJNA605907	SRR11059944	BALF	94.33	1904.89	2779	84.02	15.98	12.20	87.80	3.00	1.00	75.00
PRJNA605907	SRR11059945	BALF	99.21	267.75	0	0.00	0.00	0.00	0.00	0.00	0.00	0.00
PRJNA605907	SRR11059946	BALF	99.05	5412.00	13,461	80.54	19.46	17.18	82.82	11.00	2.00	84.62
PRJNA605907	SRR11059947	BALF	94.11	7674.39	7480	76.67	23.33	21.38	78.62	59.00	14.00	80.82
PRJNA601736	SRR10903401	BALF	3.12	102.80	2	0.00	100.00	100.00	0.00	0.00	1.00	0.00
PRJNA601736	SRR10903402	BALF	9.35	429.91	13	53.85	46.15	30.77	69.23	0.00	9.00	0.00

## Data Availability

Scripts used to detect RNA editing events are available at the following GitHub link https://github.com/BioinfoUNIBA/sars-cov-2-editing (accessed on 1 October 2021).

## Supplementary Materials

The following are available online at https://www.mdpi.com/article/10.3390/genes13010041/s1, Figure S1: Gene expression of sensor genes and RNA editing in Calu-3 Total RNA samples, Figure S2: Heatmap of sensor and ISG genes in PolyA+ RNAseq data from Calu-3, Caco-2 and H1299 infected cells at three time points post-infection (4 h, 12 h and 24 h); Table S1: Statistics and RNA editing detected in clinical samples, Table S2: Annotation of detected RNA editing sites.
