# Supplementary material for: Detection of A-to-I RNA Editing in SARS-COV-2
Source: Genes (Basel). 2021 Dec 23;13(1):41. doi: 10.3390/genes13010041 (PMC8774467; doi:10.3390/genes13010041)
Supplement: Supplementary file 1 [file genes-13-00041-s001.zip › genes-1462707-supplementary/Supplementary_Files/Supplemental Information.pdf]

Supplemental Information

Supplementary Figure S1. A) Heatmap of type I interferon genes, RIG-I and TLR receptors, and APOBECs in Calu-3 Total RNA samples. B) Gene expression of type I interferon (*IFNB1*) and key sensor genes, *DDX58* (*RIG-I*) and *IFIH1* (*MDA5*), in PolyA+ RNAseq data from Calu-3, Caco-2 and H1299 infected cells at three time points post-infection (4h, 12h and 24h). C) Expression of *ADAR1 (ADAR)* and *ADAR2 (ADARB1)* genes in PolyA+ RNAseq data from Calu-3, Caco-2 and H1299 infected cells at three time points post-infection (4h, 12h and 24h). D) Enrichment of unique hyper editing positions across infected cell lines (Calu-3, Caco-2 and H1299). E) Alu Editing Index (AEI) in all cell lines (Calu-3, Caco-2 and H1299).
Dotted lines and bars on each dot indicate mean gene expression or AEI ± SD. Values in the heatmap are $\log_2$ of normalized counts from DESeq2.

Supplementary Figure S2. Heatmap of specific genes in PolyA+ RNAseq data from Calu-3, Caco-2 and H1299 infected cells at three time points post-infection (4h, 12h and 24h). A) Heatmap of type I interferon genes, RIG-I and TLR receptors, and APOBECs in Calu-3 cells. B) Heatmap of type I interferon genes, RIG-I and TLR receptors, and APOBECs in Caco-2 cells. C) Heatmap of type I interferon genes, RIG-I and TLR receptors, and APOBECs in H1299 cells.
Several genes in B and C have been omitted because the number of supporting reads was less than 10. Values in heatmaps are $\log_2$ of normalized counts from DESeq2.

Supplementary Table S1. Statistics and RNA editing detected in clinical samples. We report the number of reads, the viral genome coverage and depth, the number of RNA variants per samples as well as the number of hyper-edited reads.

Supplementary Table S2. List of detected A-to-I RNA editing. For each site we provide the genomic location on the viral genome as well as relevant details as the inclusion in coding sequences and the potential aminoacid change. Column names are according to ANNOVAR.
