# Supplementary material for: Detection of A-to-I RNA Editing in SARS-COV-2
Source: Genes (Basel). 2021 Dec 23;13(1):41. doi: 10.3390/genes13010041 (PMC8774467; doi:10.3390/genes13010041)
Supplement: Supplementary file 1 [file genes-13-00041-s001.zip › genes-1462707-supplementary/Supplementary_Files/Supplementary_Table_S1.pdf]

BioProject	Run	Source	ReadType	ReadLen	TotalReads	FilteredRead	UniqueSars-(	Virus load	ErrorRate	Depth	Coverage	StrandOrient	AC	AG	AT	CA	CG	CT	GA	GC	GT	TA	TC	TG	ALL	A-to-I	noA-to-I	C-to-U	noC-to-U	Hyper-A-to-I	Hyper-NO-A-to-I	%Hyper
PRJNA616446	SRR11454606	Throat swab	PAIRED	117,03	11336944	8464368	2892	0,03416676	0,002144	9,64160787	0,96455205	unstranded	0	0	0	0	0	0	0	0	0	0	0	0	0	0	0	0	0	0	0	0
PRJNA616446	SRR11454607	Faeces	PAIRED	134,32	8814872	7524910	18324	0,24351122	0,0023	62,5317861	0,99692339	unstranded	0	1	0	0	0	0	0	0	0	0	0	0	1	100	0	0	100	0	0	0
PRJNA616446	SRR11454608	Throat swab	PAIRED	129,13	5380820	4816276	157470	3,26953854	0,00017	610,545932	0,99983279	unstranded	0	7	1	0	0	7	1	0	0	0	5	0	21	57,1428571	42,8571429	38,0952381	61,9047619	0	0	0
PRJNA616446	SRR11454612	Sputum	PAIRED	150,81	7510502	4880934	4024	0,08244324	0,0003	13,7615289	0,96227803	unstranded	0	0	0	0	0	0	0	0	0	0	0	0	0	0	0	0	0	0	0	0
PRJNA616446	SRR11454613	BALF	PAIRED	150,71	10837378	7759910	646234	8,32785432	0,000258	2256,34017	0,99956526	unstranded	4	818	11	1	4	80	79	3	2	9	824	1	1836	89,4335512	10,5664488	8,66013072	91,3398693	4	0	100
PRJNA616446	SRR11454614	BALF	PAIRED	150,86	11517258	7344558	1350838	18,3923662	0,000266	4167,78203	0,99969903	unstranded	13	2599	31	13	9	430	392	8	26	27	2714	8	6270	84,7368421	15,2631579	13,1100478	86,8899522	2	0	100
PRJNA616446	SRR11454615	BALF	PAIRED	150,56	10458618	7721418	93306	1,20840498	0,000304	321,725813	0,99889643	unstranded	1	5	1	0	1	3	2	0	0	0	4	1	18	50	50	27,7777778	72,2222222	0	0	0
PRJNA605907	SRR11059940	BALF	PAIRED	150,62	5894	5386	5136	95,3583364	0,000388	21,0624686	0,9894325	unstranded	0	0	0	0	0	0	0	0	1	0	0	0	1	0	100	0	100	0	0	0
PRJNA605907	SRR11059941	BALF	PAIRED	150,59	1324	544	356	65,4411765	0,000282	1,42453934	0,56281978	unstranded	0	0	0	0	0	0	0	0	0	0	0	0	0	0	0	0	0	0	0	0
PRJNA605907	SRR11059942	BALF	PAIRED	150,73	138434	129026	120716	93,5594376	0,000434	478,335786	0,875464	unstranded	0	127	0	1	4	9	11	0	2	3	129	0	286	89,5104895	10,4895105	6,99300699	93,006993	1	4	20
PRJNA605907	SRR11059943	BALF	PAIRED	150,69	15732	11784	10322	87,5933469	0,000466	39,3937398	0,34153764	unstranded	0	0	1	1	0	0	1	0	1	1	0	0	5	0	100	20	80	3	0	100
PRJNA605907	SRR11059944	BALF	PAIRED	101	899308	798700	753452	94,334794	0,0003	1904,88941	0,99969903	unstranded	2	1167	20	16	19	192	147	9	18	17	1168	4	2779	84,0230299	15,9769701	12,1986326	87,8013674	3	1	75
PRJNA605907	SRR11059945	BALF	PAIRED	101	151472	108824	107966	99,211571	0,000552	267,753804	0,86964519	unstranded	0	0	0	0	0	0	0	0	0	0	0	0	0	0	0	0	0	0	0	0
PRJNA605907	SRR11059946	BALF	PAIRED	101	5163220	4690828	4646334	99,0514681	0,000514	5411,99629	0,99993312	unstranded	24	5356	72	22	41	1109	1203	43	21	73	5485	12	13461	80,5363643	19,4636357	17,1755442	82,8244558	11	2	84,61538462
PRJNA605907	SRR11059947	BALF	PAIRED	101	55220536	53050982	49924022	94,1057453	0,000324	7674,38652	0,99093736	unstranded	4	2693	19	16	33	817	782	15	29	28	3042	2	7480	76,671123	23,328877	21,3770053	78,6229947	59	14	80,82191781
PRJNA601736	SRR10903401	BALF	PAIRED	150,5	953264	800788	24946	3,11518155	0,000082	102,800087	0,99939805	unstranded	0	0	0	0	0	1	1	0	0	0	0	0	2	0	100	100	0	0	1	0
PRJNA601736	SRR10903402	BALF	PAIRED	150,57	1353388	1120090	104712	9,34853449	0,000092	429,913989	0,99926429	unstranded	0	5	0	0	1	4	0	0	0	1	2	0	13	53,8461538	46,1538462	30,7692308	69,2307692	0	9	0
