# Supplementary figures and images for: Detection of A-to-I RNA Editing in SARS-COV-2
Source: Genes (Basel). 2021 Dec 23;13(1):41. doi: 10.3390/genes13010041 (PMC8774467; doi:10.3390/genes13010041)

# Calu-3 Total RNA

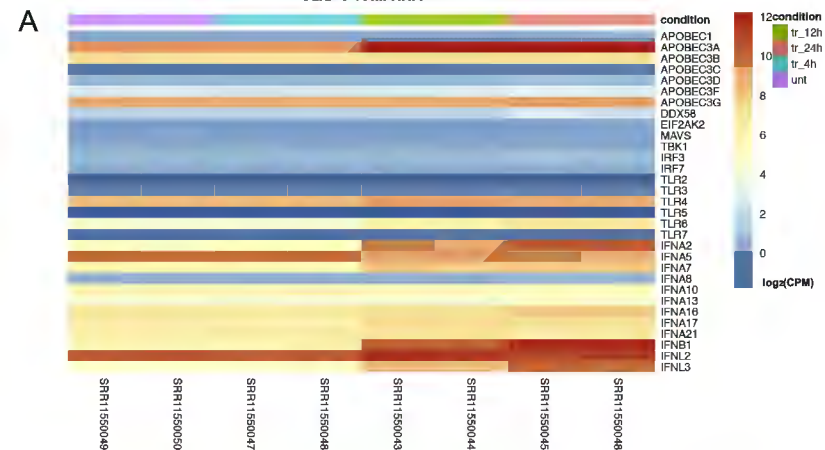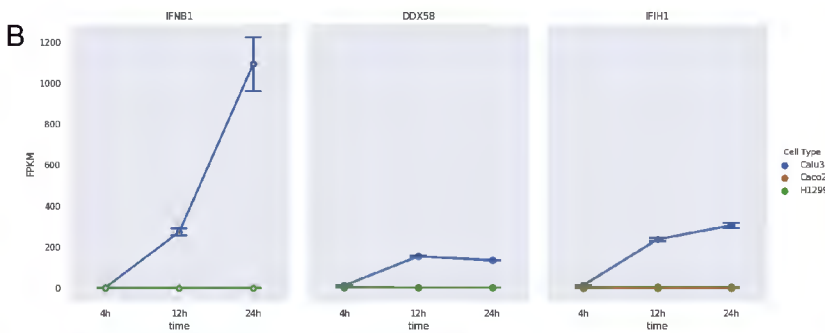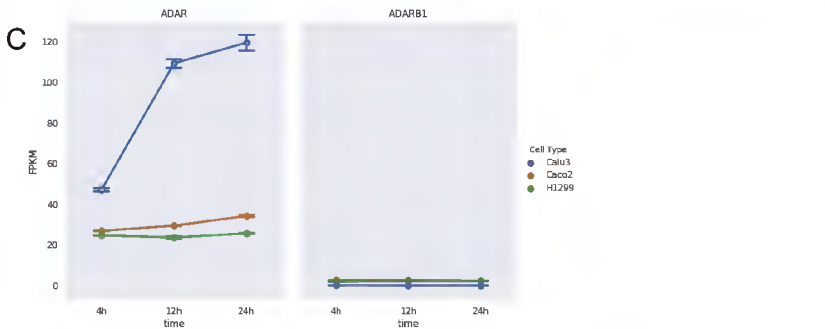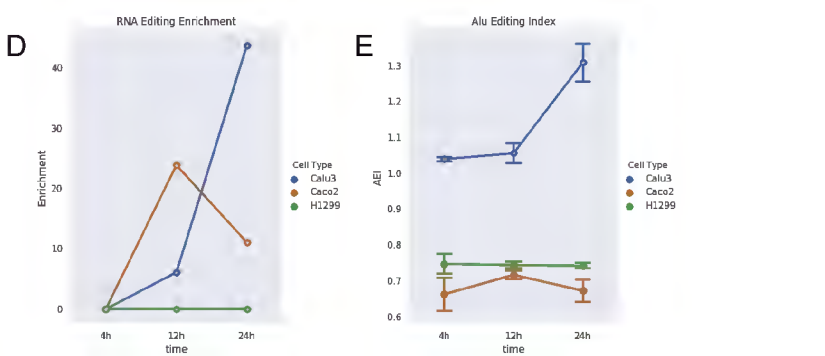

Supplement: Supplementary file 1 [file genes-13-00041-s001.zip › genes-1462707-supplementary/Supplementary_Files/Supplementary_Figure_S1.pdf]

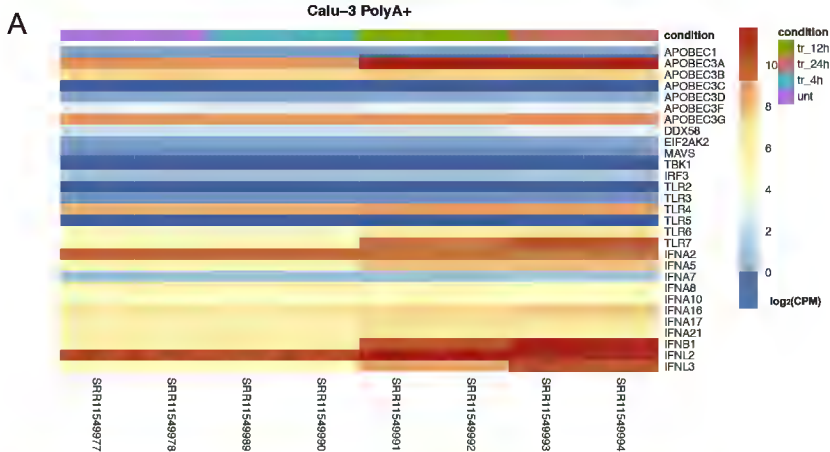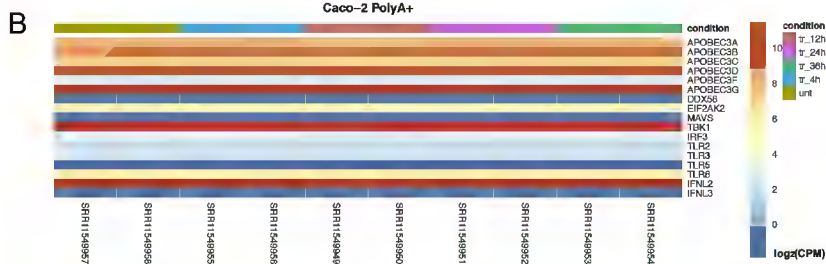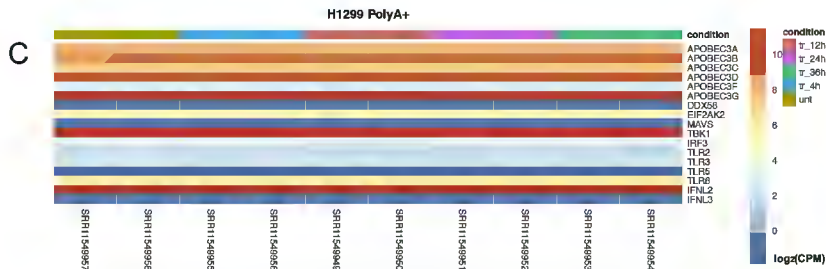

Supplement: Supplementary file 1 [file genes-13-00041-s001.zip › genes-1462707-supplementary/Supplementary_Files/Supplementary_Figure_S2.pdf]
